# Thrombus-in-Transit Across a Patent Foramen Ovale: Echocardiographic Recognition and Multimodality Management of a Fatal Paradoxical Event

**DOI:** 10.7759/cureus.116664

**Published:** 2026-09-21

**Authors:** Archana Ramalingam, Arshiya Shabnam, Bruno De Souza Goncalves, Aditya Mansabdar, Dhaval Shah

**Affiliations:** 1 Internal Medicine, Marshall University Joan C. Edwards School of Medicine, Huntington, USA; 2 Internal Medicine, New York Medical College/St. Clare’s, Denville, USA; 3 Surgery, Marshall University Joan C. Edwards School of Medicine, Huntington, USA; 4 Cardiology, New York Medical College/St. Clare's, Denville, USA

**Keywords:** cardiac surgery, echocardiography, paradoxical embolism, patent foramen ovale, pulmonary embolism

## Abstract

Thrombus-in-transit (TIT) across a patent foramen ovale (PFO) is a rare but life-threatening phenomenon that can result in paradoxical systemic embolism. Simultaneous deep venous thrombosis, pulmonary embolism (PE), and an intracardiac shunt create a critical scenario requiring rapid recognition. We report an 83-year-old woman with recurrent venous thromboembolism who presented with progressive dyspnea, pleuritic chest pain, and lower-extremity swelling. Laboratory evaluation revealed leukocytosis of 13.6 ×10⁹/L, thrombocytopenia of 67 ×10⁹/L, creatinine of 1.42 mg/dL, lactate of 2.57 mmol/L, and D-dimer of 32.53 µg/mL. Arterial blood gas analysis showed a pH of 7.47, pCO₂ of 23 mmHg, and pO₂ of 57.8 mmHg. CT pulmonary angiography demonstrated extensive bilateral pulmonary emboli with a left atrial filling defect, while echocardiography revealed a large mobile thrombus crossing a PFO with severe right ventricular dysfunction. The patient underwent emergent pulmonary embolectomy with PFO closure and left atrial appendage ligation but developed refractory cardiogenic shock and multiorgan failure despite venoarterial extracorporeal membrane oxygenation support and died 48 hours later. This case emphasizes the critical role of multimodality imaging in rapidly identifying TIT across a PFO and guiding time-sensitive multidisciplinary management.

## Introduction

Thrombus-in-transit (TIT) through a patent foramen ovale (PFO) is a rare but potentially life-threatening condition in which a venous thrombus crosses into the arterial system via an intracardiac shunt [1,2]. Although PFOs occur in one-quarter of the population, they are usually silent. Acute pulmonary embolism (PE) and elevated right atrial pressure may promote thrombus passage and systemic embolization [3,4]. Symptoms range from dyspnea and syncope to stroke or limb ischemia [5-7]. Despite its rarity, TIT across a PFO carries a substantial risk of mortality and serious systemic embolic complications, particularly in patients with massive PE, right ventricular pressure overload, or hemodynamic instability. The presence of a mobile thrombus traversing the interatrial septum also creates an immediate risk of paradoxical embolization to the cerebral, coronary, or peripheral circulation. Because of the limited number of reported cases, there are no standardized treatment guidelines, and the optimal approach remains uncertain. Management is therefore individualized and may include anticoagulation, thrombolysis, catheter-based intervention, or surgical thrombectomy depending on the patient’s hemodynamic status, thrombus characteristics, and embolic risk. Echocardiography, particularly transesophageal imaging, is central to diagnosis and assessment [8-10]. This report describes a case of TIT across a PFO in massive PE, emphasizing diagnostic and therapeutic challenges.

## Case presentation

An 83-year-old woman with a remote history of PE (treated with warfarin in 2003) and recurrent deep vein thrombosis (2019) presented with progressive dyspnea and lower-extremity swelling. Her symptoms gradually worsened over several weeks and acutely deteriorated in the three days before admission. She denied fever, cough, or hemoptysis but reported pleuritic chest pain and generalized fatigue. Given her history of recurrent venous thromboembolism, the combination of progressive dyspnea, pleuritic chest pain, lower-extremity swelling, and acute clinical deterioration raised concern for recurrent PE.

On presentation, her blood pressure was 127/80 mmHg, heart rate 83 bpm, respiratory rate 24/min, and oxygen saturation 95% on 4 L/min oxygen. Physical examination revealed jugular venous distension, bilateral pitting edema, and tenderness of the right calf. Laboratory testing showed leukocytosis (13.6 ×10⁹/L), thrombocytopenia (67 ×10⁹/L), elevated creatinine (1.42 mg/dL), and elevated lactate (2.57 mmol/L). D-dimer was markedly increased (32.53 µg/mL). Arterial blood gas analysis demonstrated hypoxemic respiratory alkalosis (pH 7.47, pCO₂ 23 mmHg, pO₂ 57.8 mmHg), consistent with ventilation-perfusion mismatch (Table 1). The combination of right calf tenderness, jugular venous distension, hypoxemia, markedly elevated D-dimer, and a prior history of venous thromboembolism further supported the clinical suspicion of an acute thromboembolic event.

**Table 1:** Laboratory Data on Admission DVT: deep vein thrombosis

Parameter	Result	Normal Range	Reference / Comment
Blood Pressure	127/80 mmHg	-	Stable on admission
Heart Rate	83 bpm	60-100 bpm	Normal sinus rhythm
Respiratory Rate	24/min	12-20/min	Mild tachypnea
Oxygen Saturation	95% (on 4 L/min O₂)	≥95%	Supplemental oxygen required
Temperature	Afebrile	-	-
Jugular Venous Distension	Present	Absent	Suggestive of elevated right atrial pressure
Lower-Extremity Edema	Bilateral, pitting	Absent	Consistent with venous congestion
Right Calf Tenderness	Present	Absent	Clinical sign of DVT
White Blood Cell Count	13.6 ×10⁹/L	4.0-11.0 ×10⁹/L	Elevated
Platelet Count	67 ×10⁹/L	150-450 ×10⁹/L	Thrombocytopenia
Creatinine	1.42 mg/dL	0.6-1.2 mg/dL	Mild renal impairment
Lactate	2.57 mmol/L	0.5-2.2 mmol/L	Elevated
D-dimer	32.53 µg/mL	<0.50 µg/mL	Markedly elevated
Arterial pH	7.47	7.35-7.45	Alkalemia
pCO₂	23 mmHg	35-45 mmHg	Decreased
pO₂	57.8 mmHg	80-100 mmHg	Hypoxemia

Computed tomography pulmonary angiography (CTPA) revealed extensive bilateral pulmonary emboli involving the main and lobar arteries, with evidence of peripheral pulmonary infarctions and a filling defect within the left atrium (Figure 1). Transthoracic echocardiography (TTE) demonstrated a large, mobile thrombus extending from the right atrium through the PFO into the left atrium, intermittently prolapsing into the left ventricle. The right ventricle was markedly dilated and hypokinetic (right ventricular systolic pressure (RVSP) 71 mmHg), confirming acute cor pulmonale (Figure 2). The simultaneous presence of extensive pulmonary embolism marked right ventricular dysfunction, and a highly mobile thrombus traversing the interatrial septum established the diagnosis of thrombus-in-transit through a PFO and indicated a substantial risk of paradoxical systemic embolization.

**Figure 1:**
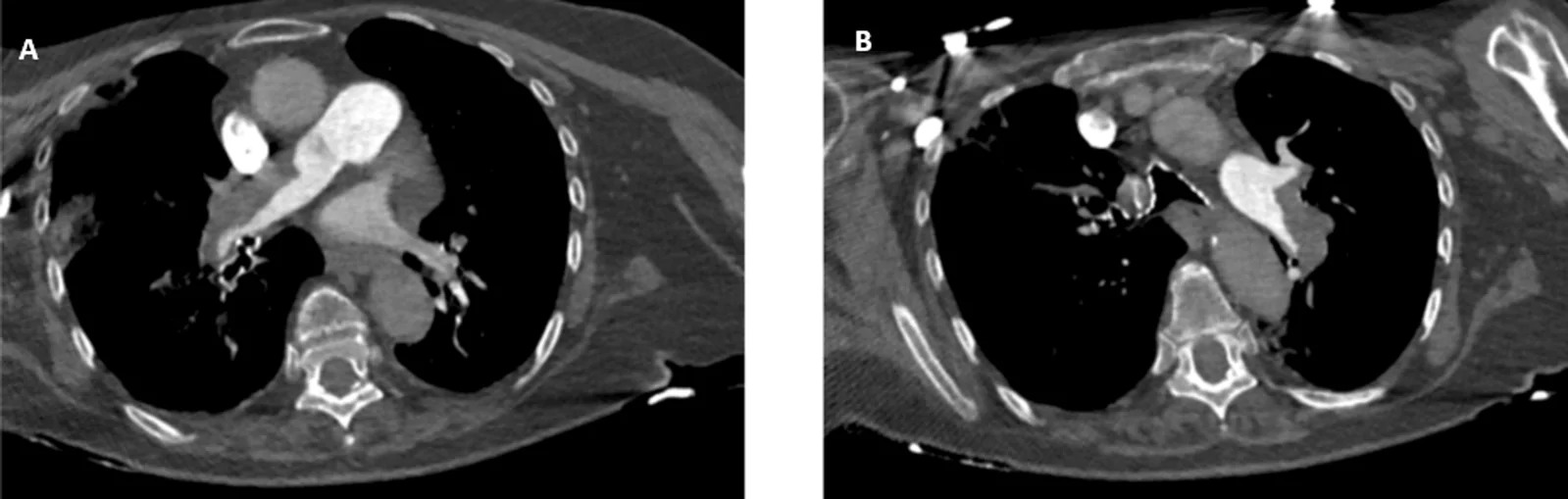
CT pulmonary angiography demonstrating extensive bilateral pulmonary emboli with a left atrial filling defect consistent with thrombus-in-transit.

**Figure 2:**
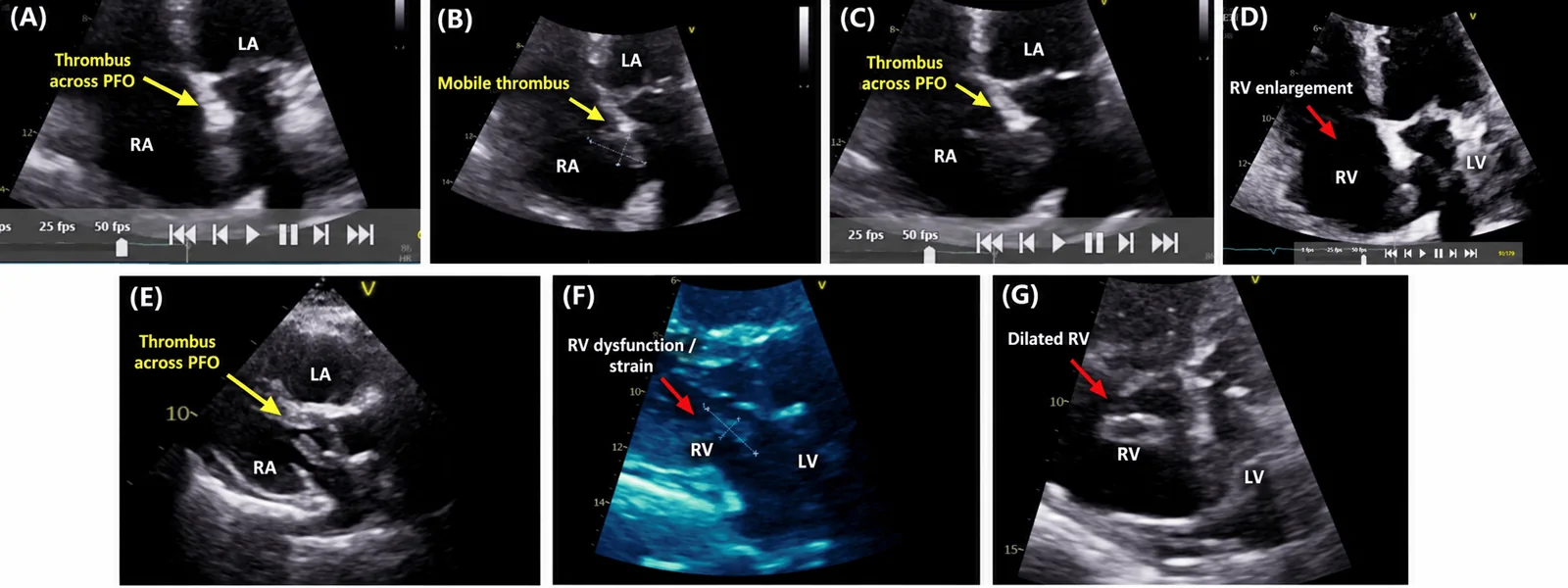
Transthoracic echocardiography shows a large, mobile thrombus crossing the patent foramen ovale with significant right ventricular dilation and hypokinesia. LA: left atrium, LV: left ventricle, PFO: patent foramen ovale, RA: right atrium, RV: right ventricle

Chronologically, the patient experienced progressive symptoms over several weeks, followed by marked clinical worsening during the three days before admission. After hospital presentation, CTPA identified extensive bilateral pulmonary embolism, and subsequent echocardiography demonstrated the thrombus traversing the PFO with severe right ventricular dysfunction. These findings prompted urgent multidisciplinary evaluation and surgical intervention.

Due to the presence of a large mobile thrombus and severe right ventricular dysfunction, an urgent multidisciplinary discussion between cardiology, cardiothoracic surgery, and critical care teams was held. The consensus favored immediate surgical embolectomy. The patient underwent open pulmonary embolectomy with removal of thrombi from both atria and ventricles, followed by surgical closure of the PFO and ligation of the left atrial appendage (LAA). Postoperatively, she required venoarterial extracorporeal membrane oxygenation (ECMO) for refractory cardiogenic shock and persistent hypoxemia. Despite aggressive management, she developed progressive multiorgan failure and died 48 hours later.

## Discussion

TIT across a PFO represents a rare and highly fatal manifestation of venous thromboembolism, linking PE and systemic embolization through an intracardiac shunt [11]. This phenomenon occurs when elevated right atrial pressures, typically secondary to acute PE or right ventricular dysfunction, reverse the normal interatrial pressure gradient, allowing a thrombus to cross the PFO into the left atrium [12,13]. Once this communication becomes functional, even transiently, systemic embolization to the brain, coronary, or peripheral arteries may occur, resulting in severe clinical consequences [14]. In this patient, systemic blood pressure was preserved at presentation (127/80 mmHg), and therefore she did not initially meet criteria for hemodynamically unstable high-risk PE. However, the extensive bilateral clot burden, marked right ventricular dilation and hypokinesis, and an estimated RVSP of 71 mmHg indicated severe right-heart strain and a clinically high-risk presentation. A more precise intermediate-risk classification was limited by the absence of complete biomarker-based risk stratification data.

The pathophysiologic cascade underlying TIT reflects a state of acute right-heart strain, where pulmonary vascular obstruction and hypoxemia cause rapid deterioration of hemodynamics. The combination of reduced left ventricular preload and impaired oxygenation can precipitate cardiogenic shock and multiorgan failure if not promptly addressed. In the present case, severe right ventricular dysfunction and hypoxemia were directly observed, whereas the contribution of altered intracardiac pressure gradients and reduced left ventricular preload represents a likely pathophysiologic mechanism rather than a directly measured finding. The risk of systemic embolization is particularly high when the thrombus is large, mobile, and partially lodged within the interatrial septum, a finding often referred to as an “impending paradoxical embolism” [15].

Echocardiography is indispensable in both diagnosis and management. TTE allows rapid bedside detection of right ventricular dilation, elevated pulmonary pressures, and the presence of an intracardiac thrombus. However, transesophageal echocardiography (TEE) remains the gold standard due to its superior image resolution, enabling precise visualization of thrombus morphology, interatrial septal anatomy, and the dynamic passage of thrombotic material between atria [16]. Intracardiac echocardiography (ICE) can serve as a valuable alternative in unstable patients or during percutaneous procedures. Additionally, advanced imaging modalities such as cardiac MRI or CT angiography can provide complementary insights into thrombus burden and right-heart function [5,17].

The optimal management of TIT remains controversial, largely due to its rarity and the absence of standardized guidelines. Anticoagulation alone may be appropriate in stable patients with small, non-mobile thrombi but carries a persistent risk of embolization. Systemic thrombolysis can be considered in hemodynamically unstable patients who are poor surgical candidates, though it increases the likelihood of thrombus fragmentation and paradoxical embolism [18,19]. Compared with anticoagulation alone, thrombolysis may provide more rapid clot reduction but may also destabilize a mobile thrombus, increasing the risk of fragmentation and systemic embolization. Surgical embolectomy, although more invasive, allows direct removal of the intracardiac and pulmonary thrombus while minimizing the risk of uncontrolled fragmentation and permitting simultaneous closure of the PFO. In the present case, the large and highly mobile thrombus traversing the PFO, together with severe right ventricular dysfunction and the risk of imminent paradoxical embolization, favored surgical intervention over anticoagulation alone or systemic thrombolysis. Surgical embolectomy with simultaneous PFO closure remains the preferred approach for large or mobile thrombi, as it allows direct clot extraction and definitive elimination of the interatrial shunt. Intraoperative exclusion or LAA may further reduce the risk of recurrent embolic events, particularly in patients with atrial stasis or dilation.

Despite advances in diagnostic imaging and surgical techniques, the prognosis of TIT across a PFO remains guarded. Mortality rates range from 18% to 45%, depending on patient age, hemodynamic status, and timing of intervention [15,20]. Although detailed intraoperative hemodynamic measurements were not available, the postoperative requirement for venoarterial ECMO for refractory cardiogenic shock and persistent hypoxemia documents severe circulatory and respiratory compromise following surgery. This distinction is important, as the patient was initially normotensive but subsequently developed profound postoperative hemodynamic deterioration. Early echocardiographic identification, rapid multidisciplinary coordination, and timely surgical management are critical determinants of survival. This case reinforces the necessity of maintaining a high index of suspicion in patients presenting with acute PE and unexplained hypoxemia, as prompt detection and intervention can be lifesaving.

## Conclusions

TIT across a PFO constitutes a rare but highly fatal manifestation of venous thromboembolism, demanding prompt recognition and decisive management. Echocardiography remains the cornerstone of diagnosis, enabling real-time visualization of thrombus morphology, interatrial communication, and hemodynamic compromise. In this case, CTPA and echocardiography provided complementary information regarding pulmonary thrombus burden, right ventricular dysfunction, and intracardiac thrombus extension, supporting rapid clinical decision-making. Optimal outcomes depend on a rapid, multidisciplinary approach integrating cardiology, cardiothoracic surgery, and critical care expertise. Close coordination among these specialties was essential in determining the need for urgent surgical intervention. The use of multimodality imaging can guide timely intervention, while preventive measures such as LAA exclusion may be considered in selected patients; however, this single case does not allow conclusions regarding its effectiveness in preventing recurrence or systemic embolization, and its role in this setting remains uncertain. Establishing standardized diagnostic pathways and management protocols will be crucial to improving survival and advancing care in this rare but devastating condition.
